# Effects of Chlorogenic Acids on Menopausal Symptoms in Healthy Women: A Randomized, Placebo-Controlled, Double-Blind, Parallel-Group Trial

**DOI:** 10.3390/nu12123757

**Published:** 2020-12-07

**Authors:** Yuka Enokuchi, Atsushi Suzuki, Tohru Yamaguchi, Ryuji Ochiai, Masakazu Terauchi, Kiyoshi Kataoka

**Affiliations:** 1 Health & Wellness Products Research Laboratories, Kao Corporation, Tokyo 131-8501, Japan; yamaguchi.tohru@kao.com; 2 Department of Research & Development, Kao Corporation, Tokyo 131-8501, Japan; suzuki.atsushi2@kao.com; 3 Biological Science Research Laboratories, Kao Corporation, Tokyo 131-8501, Japan; ochiai.ryuuji@kao.com; 4 Department of Woman’s Health, Tokyo Medical and Dental University, Tokyo 113-8510, Japan; teragyne@tmd.ac.jp; 5 Personal Health Care Products Research Laboratories, Kao Corporation, Tokyo 131-8501, Japan; kataoka.kiyoshi@kao.com

**Keywords:** chlorogenic acid, hot flushes, menopausal symptoms

## Abstract

A reduction in estrogen levels in the perimenopausal and postmenopausal periods causes various symptoms in women, such as hot flushes, sweats, depression, anxiety, and insomnia. Chlorogenic acids (CGAs), which are phenolic compounds widely present in plants such as coffee beans, have various physiological functions. However, the effects of CGAs on menopausal symptoms are unknown. To examine the effects of CGAs on menopausal symptoms, especially hot flushes, a randomized, placebo-controlled, double-blind, parallel-group trial was conducted in healthy women. Eighty-two subjects were randomized and assigned to receive CGAs (270 mg) tablets or the placebo for 4 weeks. After 4 weeks of intake, the number of hot flushes, the severity of hot flushes during sleep, and the severity of daytime sweats decreased significantly in the CGA group compared to the placebo group. The modified Kupperman index for menopausal symptoms decreased significantly after 2 weeks in the CGA group compared to the placebo group. Adverse effects caused by CGAs were not observed. The results show that continuous intake of CGAs resulted in improvements in menopausal symptoms, especially hot flushes, in healthy women.

## 1. Introduction

Peri- and postmenopausal women are afflicted by various physical and psychological disorders that are collectively referred to as menopausal symptoms, including hot flushes, sweats, insomnia, and anxiety. Hot flushes and sweats are considered vasomotor symptoms because of their vascular reactivity with vasodilation and vasoconstriction, and usually start from the face—mainly the upper body—and may spread to the head, chest, and even the whole body. Vasomotor symptoms interfere with daily activities and sleep, and can cause fatigue, loss of concentration, and symptoms of depression, affecting the quality of life (QOL) of menopausal women. Several factors have been reported to exacerbate vasomotor symptoms, for example, surgical menopause, high body mass index (BMI), and smoking.

The mechanism underlying the development of vasomotor symptoms is not fully understood. One hypothesis is that menopausal women with hot flushes have a narrower thermoneutral zone [1]. Kisspeptin/neurokinin B/dynorphin (KNDy) neurons [2], calcitonin gene-related peptide [3], vascular endothelial function [4], and autonomic nervous system function [5,6] have also been reported to be involved in the development of vasomotor symptoms. The domino theory [7] suggests that hot flushes result in reduced sleep duration, leading to the exacerbation of psychological symptoms due to a lack of sleep. Conversely, improving sleep conditions may lead to a decrease in hot flushes [8].

Various methods have been reported to relieve menopausal symptoms, and hormone replacement therapy (HRT) is accepted as the most effective treatment for hot flushes. Despite concerns about the safety of HRT after the publication of the Women’s Health Initiative (WHI) study [9], HRT has many functions in improving women’s QOL, and is considered the most effective treatment [10]. Some types of *kampo* (Japanese traditional herbal medicine) have been reported to improve menopausal symptoms [11,12], and herbs and dietary nutrients such as black cohosh [13], isoflavone [14,15], equol (a metabolite of isoflavones) [16], and proanthocyanidin [17] are also known to relieve menopausal symptoms.

Previous research has suggested that racial differences exist in the severity of hot flushes, with Asian women experiencing fewer hot flushes than Caucasian women [18]. In the equol study [16], subjects were Japanese women who experienced hot flushes approximately three times a day on average; however, dietary nutrients that reduce the relatively infrequent hot flushes may be more effective for Japanese women during menopause.

Chlorogenic acids (CGAs) are phenolic compounds widely present in plants such as coffee beans. CGAs have been documented to have antitumor [19] and antioxidant [20] effects in addition to effects in improving vascular endothelial function and hypertension [21] and reducing body fat [22]. Recent studies have reported that the continuous intake of CGAs improves autonomic nervous system activity [23] and sleep quality [24]; however, the effects on women’s menopausal symptoms are unknown. In the present study, the effects of the continuous intake of CGAs on menopausal symptoms, especially hot flushes, were examined in healthy menopausal women.

## 2. Materials and Methods

### 2.1. Study Design

A randomized, placebo-controlled, double-blind, parallel-group trial was conducted to assess the effects of CGA intake on menopausal symptoms, especially hot flushes. The total test period was 6 weeks, including a pre-observation period of 1 week (week 0), a test tablet intake period of 4 weeks (weeks 1–4), and a post-observation period of 1 week (week 5). Subjects took either 6 CGA tablets containing green coffee bean extract or 6 placebo tablets with water before sleep.

The study protocol was prepared prior to commencing the study. The study was conducted after review and approval by the Human Research Ethics Committee of Kao Corporation (approval no. T150-180720, approved 23 August 2018) and registration with the University Hospital Medical Information Network (UMIN) Clinical Trials Registry (ID: UMIN000034056). The study was conducted from September 2018 to April 2019 in accordance with the Declaration of Helsinki and directed entirely by Research and Development Inc. (Tokyo, Japan). The primary endpoint was menopausal symptoms (the number of hot flushes) at four weeks after the completion of intake, and the secondary endpoints were the number of sweats, severities of hot flushes and sweats, general menopausal symptoms, health-related QOL (HRQOL), and anxiety.

### 2.2. Subjects

Healthy female volunteers aged 40–59 years with menopausal symptoms were recruited from around the Tokyo metropolitan area. The inclusion criteria for the subjects were as follows: healthy women 40–59 years of age, moderate or severe rating according to the modified Kupperman menopausal index (mKMI) severity grading (including a moderate or a lower score in some cases), and subjective symptoms of hot flushes. The exclusion criteria were as follows: those affected by disease; taking regular medication; receiving regular outpatient treatment at a medical facility or visited a hospital within one month prior to the pre-examination; taking HRT; taking medication that may interfere with hormones; regularly taking supplements that affect menopausal symptoms; regularly taking functional foods and supplements that have been shown to be effective in improving sleep; pregnant, breastfeeding, or willing to become pregnant during the study period; smokers; unable to follow the dietary restrictions during the study period; known to have a past or current history of allergies; leading irregular lifestyles; or deemed unfit for enrollment by the physician in charge or by the principal investigator due to other causes. During the study, subjects limited their coffee intake to one cup per day. Written informed consent was obtained from all subjects before the commencement of the study.

**Figure 1:**
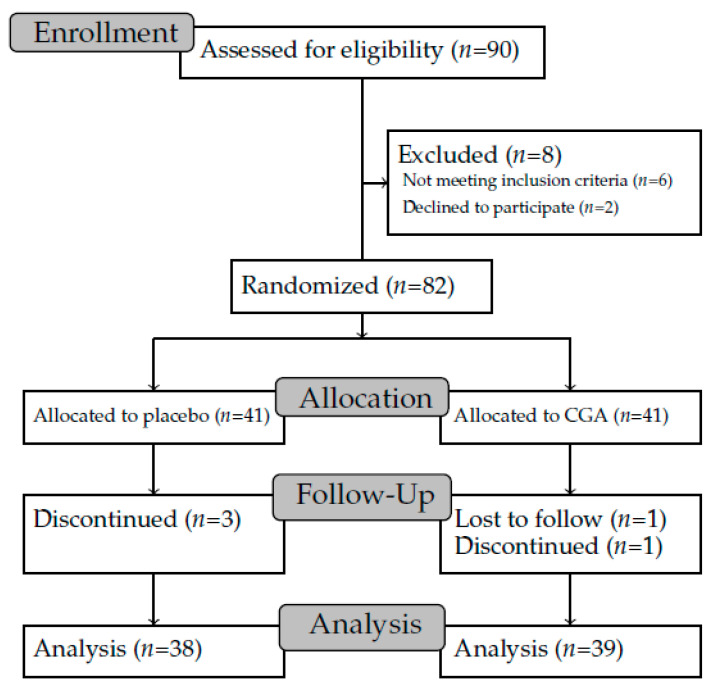
Flow diagram of the study from subject enrollment to analysis. CGA, chlorogenic acid.

Figure 1 shows the flow from subject enrollment to analysis. Of the 90 recruited subjects, 82 participated in the study. These subjects were randomly assigned to two groups, the CGA group and the placebo group, using stratified randomization to ensure that the subjects were equally distributed (*n* = 41 subjects per group) according to the number of hot flushes, severity of menopausal symptoms, age, and menstrual status as identified in the pre-survey.

Of the 82 subjects, three in the placebo group withdrew from the study (one subject was prescribed an antihypertensive drug during the pre-observation period, one subject developed symptoms of insomnia during the study period, and one subject was hospitalized due to an injury during the study period). In the CGA group, two subjects were excluded from the study (one subject discontinued the intake of the test tablets due to a cold during the study period, and one subject for personal reasons). Therefore, 77 subjects who completed study (38 subjects in the placebo group and 39 subjects in the CGA group) were included in the analysis. The mean intake rate of the test tablets among those who completed the study was 98.6% in the CGA group and 99.6% in the placebo group. For the 385 data points (corresponding to 5 weeks multiplied by 77 subjects), there were three missing measurements in the hot flush dataset and six missing measurements in the sweat dataset.

The background characteristics of the subjects who completed the study in each group are shown in Table 1. There were no significant differences between the two groups in terms of any of the items. The causal relationships of adverse events identified during and after the study was completed were evaluated by a physician. Of the four adverse events identified during the study period, the causal relationship with the study intervention of one subject in the placebo group, who developed insomnia after the start of ingestion of the study tablets, was deemed “undeniable”; however, there was no conclusion that there was a causal relationship, including the events identified after the completion of the study.

### 2.3. Test Tablets

CGAs were extracted from green coffee beans with hot water, and caffeine was removed from the extract using activated carbon. The extract was spray-dried to obtain a powder. The compositions of the CGAs were determined using high-performance liquid chromatography and were as follows: caffeoylquinic acids (CQAs) 5-caffeoylquinic acid, 3-caffeoylquinic acid, and 4-caffeoylquinic acid; feruloylquinic acids (FQAs) 3-feruloylquinic acid, 4-feruloylquinic acid, and 5-feruloylquinic acid; dicaffeoylquinic acids (di-CQAs) 3,4-dicaffeoylquinic acid, 3,5-dicaffeoylquinic acid, and 4,5-dicaffeoylquinic acid. The CGA tablets (300 mg/tablet, 6 tablets/day) were industrially manufactured to be homogeneous with excipients and flavoring so that six CGA tablets contained 270 mg of the main CGA components (CQAs and FQAs). The placebo tablets did not contain CGAs and were replaced with excipients. According to an analysis by a third-party analysis agency for manufactured tablets, the six CGA tablets contained 272.5 mg of the main components of CGAs (CQAs and FQAs), 1.35 g of carbohydrates (including CGAs), 338 mg of protein, and 34.2 mg of fat. The six placebo tablets contained 1.72 g of carbohydrates, 1.8 mg of protein, and 37.8 mg of fat. The subjects could not identify which tablets they were taking based on the appearance and taste of the tablets. 

### 2.4. Assessment of Hot Flushes and Sweats

The subjects recorded the number of hot flushes and sweats, wake-up time, and bedtime over a total period of 6 weeks (weeks 0–5), and they also recorded whether test tablets were taken during weeks 1–4 (4 weeks) in their daily diary. The severities of hot flushes and sweats during the daytime or sleep was assessed every two weeks using a visual analog scale (VAS) (last day of weeks 0, 2, and 4). The VAS was represented as a 100 mm-long straight line with the left end representing “no symptoms at all” and the right end representing “extremely severe symptoms”. Subjects marked the scale according to the severity of items, and the position was measured as the distance (mm) from the left edge.

### 2.5. Assessment of Menopausal Symptoms, HRQOL, and Anxiety

The mKMI [25] is a questionnaire that is a modification of the Kupperman menopausal index (KMI) [26] for the assessment of Japanese women. The mKMI includes 17 questions in which questions related to symptoms characteristic of Japanese menopausal women are added to the 11 questions of the original KMI. The 17 questions were classified into the following 11 symptoms according to the KMI: vasomotor, paresthesia, insomnia, nervousness, melancholia, vertigo, weakness/fatigue, arthralgia and myalgia, headaches, palpitation, and formication. For each of the 17 questions, subjects selected the level of the condition according to 4 levels (strong, medium, weak, and none) experienced in the previous 2–3 days. The highest severity for each question in the symptom group was taken as the severity of the symptom group, and the score was calculated using the KMI weighting method. The highest possible total score for the mKMI was 51, which is the same as for the KMI, and the severity grading was assessed according to the following scores: 0–12 is I, 13–22 is II, 23–33 is III, 34–43 is IV, and 44–51 is V. I and II are considered as mild, III as moderate, and IV and V as severe. The mKMI was used to evaluate the effect of intervention, and determining the severity was not its primary purpose. We used the mKMI to confirm the effects of the intervention.

The Short Form-8 (SF-8) is a scale that can measure 8 areas of health and HRQOL measures [27]. From SF-8, the Physical Component Summary (PCS-8) score and Mental Component Summary (MCS-8) score were calculated. There are 8 items in the SF-8, and the appropriate SF-36 v2 subscale score is assigned to the category of answers for each item. The subscale of SF-8 is weighted and added by the coefficient for PCS-8 or the coefficient for MCS-8 (coefficient of the regression obtained from Japanese general population data). By adding an intercept of the regression, the scores of PCS-8 and MCS-8 are standardized in the Japanese national data so that they have the same meaning as the summary score of SF-36. The higher the SF-8 score, the better the health condition. The State-Trait Anxiety Inventory (STAI) is a psychological test where the degree of state anxiety and trait anxiety are each scored from a different set of 20 answers [28]. The higher the scores for state and trait anxiety, the greater the anxiety. In this study, we used the standard version of the STAI, in which the past month was considered as the review period.

The study began on a Friday, and subjects performed the assessments on the designated Thursday nights. The mKMI was conducted on the last day of weeks 0, 2, and 4. The SF-8 and STAI were administered on the last day of weeks 0 and 4. 

### 2.6. Statistical Analysis

To estimate the sample size, we referred to previous studies that evaluated the role of dietary nutrients in the health of menopausal women. Hirose et al. investigated the effects of low-dose soy isoflavone aglycone in 90 subjects (each group consisted of 30 subjects) [15], and Terauchi et al. examined the effects of grape seed proanthocyanidin extract in 96 subjects (each group consisted of 31–33 subjects) [17]. In this study, we set the recruitment target for each group to 45 subjects, anticipating that the improvement effect would be difficult to determine because the subjects had a relatively low frequency of hot flushes, which was the primary endpoint. 

The indicators representing the characteristics of the subjects at baseline were reported as quartiles and means ± standard deviations (SDs) for continuous variables, and categorical variables were presented as frequencies. The number of hot flushes and the number of sweats were totaled weekly at weeks 0, 1, 2, 3, and 4. The subjects with missing data measurements were excluded from the data at that time. These weekly data were summarized as means and standard errors (SEs) using the Poisson regression model as they were count values. The comparisons between the placebo and CGA groups were conducted using the model, adjusted to use week 0 as the baseline. The objective variable of this model was the number of hot flushes and sweats at week 4 as the primary and secondary endpoints, respectively; however, weeks 1, 2, and 3 were also examined. The amount of change from the baseline VAS assessment was reported as the box plot, and the Wilcoxon rank-sum test was used for comparison. The mKMI score was also presented as mean ± SE. Lastly, the data from the questionnaire on the various symptoms were compared between groups using the Wilcoxon rank-sum test. 

Statistical analyses were conducted using R statistical software and environment (version 4.0.1). All statistical analyses were double-sided. *p* < 0.05 was considered to indicate statistically significant differences.

## 3. Results

### 3.1. Number of Hot Flushes and Sweats

At week 0, the weekly mean ± SD of the number of hot flushes in the subjects was 9.4 ± 10.8 in the placebo group (*n* = 38) and 10.2 ± 11.6 in the CGA group (*n* = 39). The weekly average of the entire analytical dataset (*n* = 77) was 9.8 ± 11.1, which indicates that the daily mean for hot flushes at the start of the study was 1.4 ± 1.6. In week 4, the average weekly hot flushes decreased to 4.2 ± 7.6 in the placebo group and 3.7 ± 5.1 in the CGA group. The frequency of sweats also decreased from 6.1 ± 6.8 (week 0) to 2.8 ± 4.5 (week 4) in the placebo group and from 9.6 ± 10.8 (week 0) to 3.5 ± 4.5 (week 4) in the CGA group.

**Figure 2:**
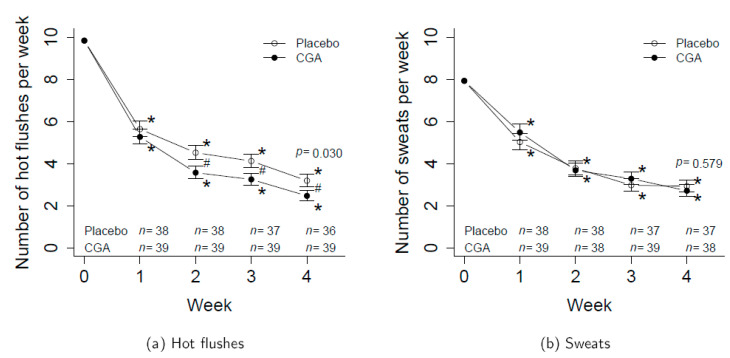
The estimated number of (**a**) hot flushes and (**b**) sweats per week (times/week). Error bars represent standard errors. *p*-values are the results of the comparison between groups at week 4. ^#^ represents *p* < 0.05 between groups. * represent *p* < 0.05 within groups. Numbers for weeks 1, 2, 3, and 4 were estimated using the Poisson regression model with adjustment of week 0 number. Participants with missing values were excluded. CGA, chlorogenic acid.

The results of the statistical analysis, after excluding those with missing data at week 4, showed a significant difference in the mean weekly number of hot flushes between the CGA and placebo groups (Figure 2a). The *p*-values between the groups in terms of the weekly number of hot flushes at week 1, 2, 3 and 4 were 0.433, 0.017, 0.022, and 0.030, and the false discovery rate (FDR)-adjusted *p*-values, i.e., *q*-values, were 0.433, 0.040, 0.040, and 0.040, respectively. The results of the subgroup analysis according to menopausal status are shown in the Supplementary Materials (Figure S1). We found no significant differences in the mean weekly number of sweats between groups (Figure 2b). The *p*-values between the group of the weekly number of sweats at week 1, 2, 3 and 4 were 0.387, 0.823, 0.451, and 0.579, and the *q*-values were 0.772, 0.823, 0.772, and 0.772, respectively. 

### 3.2. Severities of Hot Flushes and Sweats (VAS Assessment)

**Figure 3:**
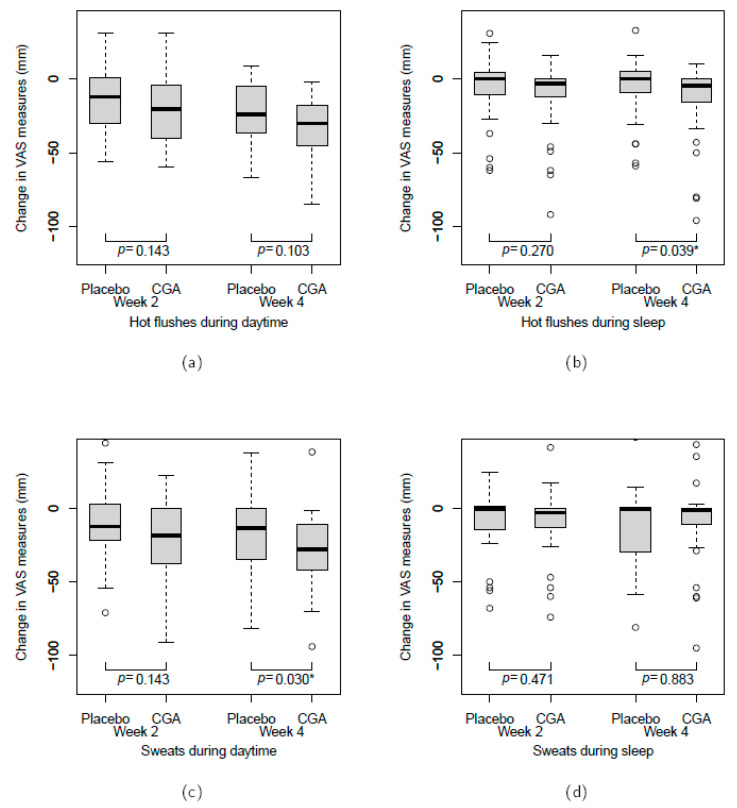
Change in the visual analogue scale (VAS) measurements of hot flushes and sweats compared to week 0: hot flushes during (**a**) the daytime and (**b**) sleep; sweats during (**c**) the daytime and (**d**) sleep. The comparison was assessed using the Wilcoxon rank-sum test. * *p* < 0.05. There was one subject in the chlorogenic acid (CGA) group with missing data for the VAS measurements at week 0. CGA, chlorogenic acid.

Figure 3 shows the amount of change in the severity of hot flushes and sweats among the subjects compared to week 0 (baseline); the data of three subjects were excluded due to recording errors in either week 0 or 4. At week 4, we found significant differences in the severity of hot flushes during sleep and daytime sweats between the placebo and CGA groups according to the Wilcoxon rank-sum test.

### 3.3. Menopausal Symptoms

**Figure 4:**
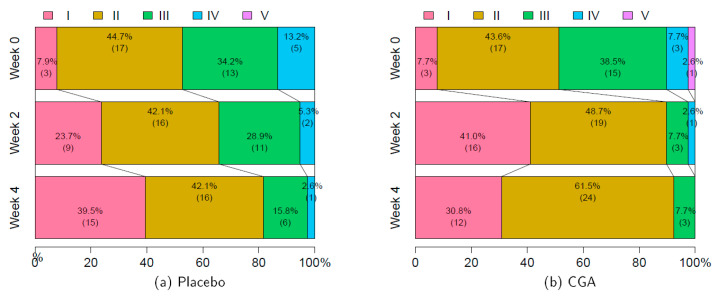
Severity grading based on the modified Kupperman menopausal index scores: (**a**) placebo and (**b**) CGA. Severity grading was assessed according to the following scores: 0–12 is I, 13–22 is II, 23–33 is III, 34–43 is IV, and 44–51 is V. I and II are considered as mild, III as moderate, and IV and V as severe. Comparisons between placebo vs. CGA were *p* = 0.906, *p* = 0.016, and *p* = 0.173 for weeks 0, 2, and 4, respectively, as assessed by a logistic regression model with a threshold score between 22 and 23. Numbers in parentheses indicate frequencies. CGA, chlorogenic acid.

Figure 4 shows the results of the mKMI severity grading. The subjects were women experiencing hot flushes who were otherwise healthy, and more than half of the subjects had a mKMI grade of I or II at week 0. Therefore, we conducted a statistical test focusing on the change in the proportion of grade I or II mKMI. Although improvements in menopausal symptoms were observed over time in both the placebo and CGA groups, the improvement was faster in the CGA group than in the placebo group, with a significant difference demonstrated between the groups at week 2 according to the Wilcoxon rank-sum test.

### 3.4. HRQOL and Anxiety

Table 2 shows the results for the SF-8 and STAI. The PCS-8 scores and MCS-5 scores of the SF-8 significantly increased compared to the initial values in both the placebo and CGA groups, and the state and trait anxiety of STAI decreased in both groups. All findings showed significant improvements compared to the initial values in both groups; however, no significant difference between the two groups was observed.

## 4. Discussion

The hot flushes that afflict menopausal women are classified as vasomotor symptoms that are strongly associated with physical and mental disorders such as insomnia and anxiety, and there are various theories regarding the mechanism underlying their development. We focused on CGAs, dietary nutrients that affect sleep, autonomic nervous system activity, and vascular endothelial function, which are the factors involved in hot flushes, and investigated whether CGAs can reduce the frequency and severity of hot flushes.

When healthy menopausal women were administered 270 mg of CGAs for four weeks, they reported a significant decrease in the number of hot flushes. The subjects in this study were women considered to be healthy except for experiencing an average of 1.4 hot flushes per day, suggesting that the continuous intake of CGAs may further improve the symptoms of relatively infrequent hot flushes. The frequency and severity of vasomotor symptoms, such as hot flushes and sweats, vary depending on the individual, and it is important to address not only the frequency but also the severity of such symptoms. In addition to affecting the QOL during the day, vasomotor symptoms have been shown to cause persistent insomnia and depressive symptoms by interfering with sleep, as suggested by the domino theory [7]. In the present study, we found a significant improvement in the CGA group, namely a reduced severity of sleep hot flushes and daytime sweats (VAS). These results suggest that the continuous intake of CGAs relieves vasomotor symptoms and contributes to improving QOL in menopausal women.

To assess the effectiveness of the continuous intake of CGAs on overall menopausal symptoms, the severity grading was assessed from the total mKMI score, and population changes were observed. The proportion of grade I or II mKMI showed a significant improvement with CGA intake in the second week. This result suggests that some menopausal symptoms may also be ameliorated by the ingestion of CGAs. 

The placebo group also showed an improvement in the frequency of hot flashes and sweats from the baseline. Menopausal symptoms have been reported to be affected by psychological status [29]. In this study, taking a test tablet may have raised the expectations of the subjects, and they may have been relieved to find out the effect of taking the tablets from the physical condition records. In the CGA group, in addition to effects of the CGAs, psychological effects also appeared; therefore, we considered that an improvement was experienced earlier in the CGA group compared to the placebo group. These results suggest that a combination of psychotherapy, behavioral therapy, and the intake of CGAs is beneficial.

Why did the continuous intake of CGAs improve menopausal symptoms, especially vasomotor symptoms? There have been many studies on vasomotor symptoms, including the involvement of sleep, autonomic activity, and vascular endothelial function. The domino theory states that vasomotor symptoms cause sleep deprivation and exacerbate psychological symptoms [7], while improving sleep status has been demonstrated to improve vasomotor symptoms [8]. Regarding the autonomic nervous system of menopausal women, Thurston et al. investigated the autonomic nervous activity of peri- and postmenopausal women using power spectrum analysis of heart rate variability [5]. A significant reduction in cardiac vagal control was found to occur during the hot flushes assessed in women’s daily activities. Other reports have observed that the sympathovagal balance index value was higher in postmenopausal women [6]. In addition, there is a report that a greater frequency of physiological hot flushes was associated with poorer endothelial function among younger midlife women [4].

CGAs have been reported to have effects on sleep, autonomic nerves, and vascular endothelial function. Ochiai et al. conducted a study in which adult men were given a drink containing 300 mg of CGAs for 2 weeks and reported reduced fatigue upon awakening and significantly improved sleep quality in the CGA group [24]. Between the groups, a significant difference in the sleep efficiency and total nocturnal awaking time in the second half of week 2 was also reported. In a study of nine healthy men and women taking a test beverage containing 600 mg of CGAs for five days, Park et al. found that CGAs shortened sleep latency compared with the control group, as well as enhanced parasympathetic activity, as assessed using heart rate variability during sleep [30]. 

In addition, Kagawa et al. conducted a study in which 10 healthy men were given a beverage containing 270 mg of CGAs for 4 weeks, and the analysis of heart rate variability showed that CGAs significantly increased parasympathetic nervous activity and decreased sympathetic nervous activity [23]. Based on the above results, the continuous intake of CGAs was expected to enhance parasympathetic nerve activity, improve sleep quality, and reduce daytime fatigue. The continuous intake of CGAs has also been reported to alleviate high blood pressure [31] and improve cutaneous blood flow regulation after cold stress [32]. 

CGAs are known to have oxygen-scavenging [33], endothelial nitric oxide synthase-activating [34], and nicotinamide adenine dinucleotide phosphate oxidase-inhibiting [35] effects. These effects suggest that CGA improves the bioavailability of nitric oxide in vascular endothelial cells. Therefore, the effects of CGAs on sleep, autonomic nerves, and vascular endothelial function appear to result in improved vasomotor symptoms.

CGAs are widely found in foods, such as coffee beans, apples, pears, tomatoes, potatoes, and eggplants. Of these foods, coffee is rich in CGAs, e.g., single cup of coffee contains 27–121 mg of CGAs [36]. However, coffee also contains caffeine, which exacerbates menopausal symptoms [37]. Therefore, we developed high concentrated CGA tablets, as the form of a tablet may be useful in terms of efficient and convenient intake of CGAs.

Methods for improving menopausal symptoms include HRT and the consumption of medications, such as Japanese traditional herbal medicines (*kampo*), herbs such as black cohosh, and dietary nutrients such as isoflavone, equol, and proanthocyanidin. The present findings show that the continuous intake of CGAs, which are a polyphenol found in coffee and other foods, relieved hot flushes in healthy women. Accordingly, the results of our study show that the consumption of CGAs is a potential method for improving menopausal symptoms.

The limitations of this study include the subject population being limited to those with mild symptoms, no objective assessment of sleep, and lack of measurement of autonomic nervous system activity and vascular function. To further determine the mechanism through which menopausal symptoms of healthy women are improved by CGA intake, a diverse range of subjects should be recruited in future studies, with monitoring of menopausal symptoms and measurement of sleep, autonomic nervous system activity, and vascular function.

## 5. Conclusions

The continuous intake of CGAs appears to result in improvement in menopausal symptoms, especially hot flushes, in healthy Japanese women.

## Figures and Tables

**Table 1:** Baseline characteristics by treatment group (per protocol set).

	Placebo	CGA	Test Statistic
	*n* = 38	*n* = 39	
Age (years)	48.2, **51.0**, 55.0 (51.4 ± 4.3)	48.0, **51.0**, 54.5 (51.3 ± 4.6)	$F_{1.75}=0,\;p=0.992^{\;1}$
Height (cm)	155.0, **159.5**, 162.0 (158.4 ± 5.0)	155.5, **158.0**, 162.0 (158.5 ± 4.7)	$F_{1.75}=0.02,\;p=0.899{\;}^{1}$
Body weight (kg)	49.2, **54.5**, 60.0 (55.0 ± 7.2)	51.0, **55.0**, 59.0 (54.9 ± 6.9)	$F_{1.75}=0.01,\;p=0.932^{\;1}$
BMI (kg/m^2^)	19.6, **21.7**, 23.7 (22.0 ± 3.1)	19.9, **21.3**, 23.2 (21.9 ± 2.8)	$F_{1.75}=0.03,\;p=0.872^{\;1}$
mKMI total score	17.2, **22.0**, 27.8 (23.1 ± 8.0)	16.0, **22.0**, 27.5 (22.5 ± 8.0)	$F_{1.75}=0.12,\;p=0.731{\;}^{1}$
Menopause Status			$\chi_{3}^{2}=1.25,\;p=0.742{\;}^{2}$
Premenopausal	16% (6)	15% (6)	
Perimenopausal	32% (12)	36% (14)	
Postmenopausal	53% (20)	46% (18)	
Missing	0% (0)	3% (1)	

The three numbers left **center** right represent the lower quartile left, the median **center**, and the upper quartile right for continuous variables. x ± s represents $\bar{X}\pm 1\;\mathrm{SD}$. Numbers in parentheses after percentages indicate frequencies. Tests used: ^1^ Wilcoxon rank-sum test; ^2^ Pearson’s χ^2^ test. CGA, chlorogenic acid; BMI, body mass index; mKMI, modified Kupperman menopausal index.

**Table 2:** Baseline and change of the Short Form-8 (SF-8) and State-Trait Anxiety Inventory (STAI) scores.

	Baseline		Change from Baseline at Week 4				Difference of Change
	Placebo	CGA	Placebo	*p*-Value	CGA	*p*-Value	*p*-Value
	*n* = 38	*n* = 39	*n* = 38	Within Groups	*n* = 39	Within Groups	Between Groups
SF-8							
PCS-8 score	43.4 ± 1.0	45.7 ± 1.1	2.6 ± 1.1	0.018 *	2.4 ± 1.1	0.025 *	0.712
MCS-8 score	44.6 ± 1.3	44.7 ± 1.3	4.7 ± 1.1	<0.001 *	3.5 ± 1.2	0.004 *	0.308
STAI							
State anxiety	45.7 ± 1.7	46.5 ± 1.6	−4.8 ± 1.5	0.003 *	−4.5 ± 1.3	0.002 *	0.560
Trait anxiety	50.2 ± 1.9	48.4 ± 1.7	−4.6 ± 1.1	<0.001 *	−4.0 ± 1.1	0.004 *	0.465

Summary statistics are presented as mean ± standard error. *p*-values were derived by Wilcoxon rank-sum test. * *p* < 0.05. CGA, chlorogenic acid; SF, Short Form; PCS, Physical Component Summary; MCS, Mental Component Summary; STAI, State-Trait Anxiety Inventory.

## Supplementary Materials

The following are available online at https://www.mdpi.com/2072-6643/12/12/3757/s1, Figure S1: Subgroup analysis of the estimated number of hot flushes per week (times/week).
