# Supplementary material for: Effects of Chlorogenic Acids on Menopausal Symptoms in Healthy Women: A Randomized, Placebo-Controlled, Double-Blind, Parallel-Group Trial
Source: Nutrients. 2020 Dec 7;12(12):3757. doi: 10.3390/nu12123757 (PMC7762261; doi:10.3390/nu12123757)
Supplement: Supplementary file 1 [file nutrients-12-03757-s001.pdf]

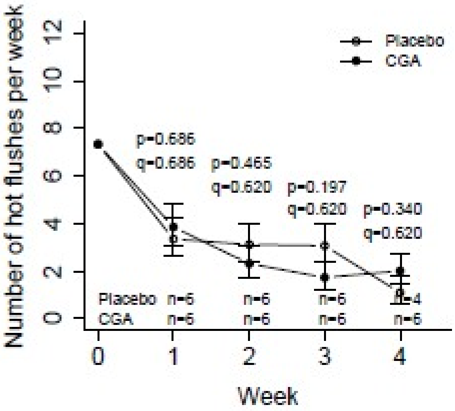

(a) Hot flushes / Premenopausal

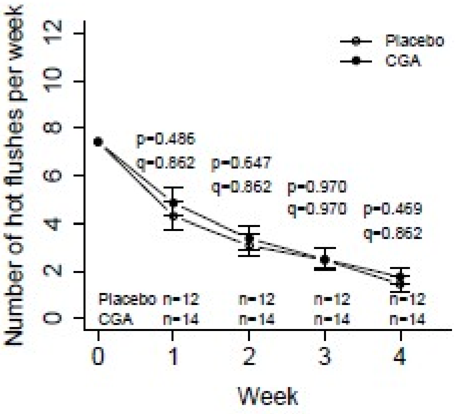

(b) Hot flushes / Perimenopausal

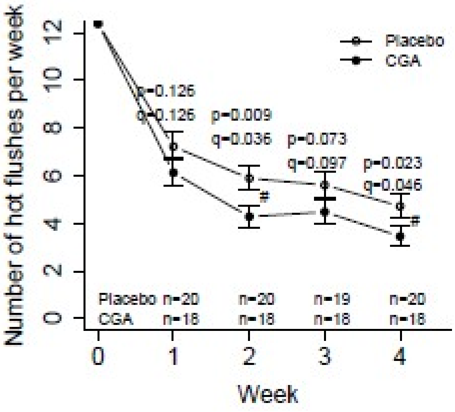

(c) Hot flushes / Postmenopausal

Figure S1. Subgroup analysis of the estimated number of hot flushes per week (times/week). Error bars represent standard errors. # represents $p < 0.05$ between groups. Numbers for Weeks 1, 2, 3, and 4 were estimated by the Poisson regression model with adjustment of Week 0 number. Participants with missing values were excluded. CGA, chlorogenic acid.
